# Supercharged End-to-Side Anterior Interosseous-to-Ulnar Motor Nerve Transfer: A Systematic Review and Meta-analysis

**DOI:** 10.1016/j.jhsg.2026.101027

**Published:** 2026-06-03

**Authors:** Abdulaziz M. Alghamdi, Salman S. Qasim, Taif Fawaz AlNojaidi, Halah Alhwsawi, Lama A. Alkhwildi, Yara K. Alwathnani, Maysaa Abdullah Alghamdi, Abdulrahman Alhadlaq, Abdulaziz K. Alhujayri

**Affiliations:** ∗ Department of Plastic and Reconstructive Surgery, Ministry of the National Guard - Health Affairs, Riyadh, Saudi Arabia; † King Abdullah International Medical Research Center, Riyadh, Saudi Arabia; ‡ College of Medicine, King Saud bin Abdulaziz University for Health Sciences, Riyadh, Saudi Arabia; § Division of Plastic and Reconstructive Surgery, King Fahad Medical City, Riyadh, Saudi Arabia; ‖ College of Medicine, Imam Mohammad Ibn Saud Islamic University (IMISU), Riyadh, Saudi Arabia; ¶ College of Medicine, University of Jeddah, Jeddah, Saudi Arabia; # Plastic Surgery Department, Security Forces Hospital, Riyadh, Saudi Arabia

**Keywords:** Anterior interosseous nerve, Cubital tunnel syndrome, Supercharged end-to-side nerve transfer, Ulnar nerve palsy

## Abstract

**Purpose:**

Ulnar nerve (UN) injuries frequently result in intrinsic hand muscle weakness and functional impairment, particularly in high-level lesions where prolonged axonal regeneration limits recovery. The supercharged end-to-side (SETS) anterior interosseous nerve (AIN)-to-ulnar motor nerve transfer was developed to enhance distal reinnervation and preserve intrinsic motor endplates. This study aimed to evaluate outcomes following SETS AIN-to-ulnar motor nerve transfer for UN pathology.

**Methods:**

A systematic review and meta-analysis were conducted in accordance with Preferred Reporting Items for Systematic reviews and Meta-Analyses guidelines. MEDLINE, PubMed, Embase, and Cochrane Central Register of Controlled Trials (CENTRAL) were searched. Studies reporting postoperative outcomes following SETS transfer for UN pathology were included. A random-effects meta-analysis was performed for binary outcomes.

**Results:**

Sixteen studies comprising 447 patients met the inclusion criteria. The pooled rate of intrinsic muscle recovery, defined as Medical Research Council (MRC) grade ≥ 3, was 80.6%. Recovery to near-normal strength (MRC ≥ 4) was achieved in 64.4%. Subgroup analysis demonstrated a notably higher MRC ≥ 4 recovery rate in traumatic injuries (85.5%) compared with compressive (52.9%) or mixed pathologies (33.3%). Surgical intervention within 12 months was associated with notably improved MRC ≥ 3 recovery. Functional outcomes improved, with mean grip strength reaching 75.7% of the contralateral side. Residual clinical signs persisted, including Froment’s sign (17.6%), ulnar clawing (12.1%), and Wartenberg’s sign (59.1%). The overall complication rate was 7.9%.

**Conclusions:**

SETS AIN-to-ulnar motor nerve transfer is a safe and effective adjunct for high UN pathology, particularly when performed early. Traumatic injuries demonstrate the highest rates of near-normal intrinsic recovery, although residual clinical signs may persist.

**Type of study/level of evidence:**

Therapeutic III.

Ulnar nerve (UN) injuries are among the most common peripheral nerve injuries affecting the upper extremities.^1^ These injuries arise from a wide spectrum of etiologies, including trauma, neuritis, brachial plexus injury, and compression neuropathies, such as cubital tunnel syndrome.^2^ Cubital tunnel syndrome is the second most common compressive neuropathy of the upper extremity and commonly results in intrinsic muscle weakness and atrophy, along with numbness and paresthesia in the ulnar distribution of the hand, ultimately leading to functional impairment and disability.^3^

The prognosis following UN injury depends on multiple factors, including lesion level, patient-related characteristics, timing of intervention, defect length, and associated injuries.^4^ Among these variables, lesion level is consistently reported as the strongest predictor of intrinsic motor recovery. Higher-level injuries are associated with poorer outcomes, with lesions proximal to the flexor digitorum profundus (FDP) demonstrating the poorest functional prognosis.^5^ This is largely attributed to the increased regeneration distance and prolonged denervation time of the intrinsic hand muscles. Traditional operative strategies include neurolysis, interpositional nerve grafting, neurorrhaphy, nerve or tendon transfers, and end-to-end anterior interosseous nerve (AIN)-to-ulnar motor nerve transfer.^6^ A major limitation of these approaches is the prolonged distance required for axonal regeneration to reach the distal motor endplates, thereby increasing the risk of irreversible motor endplate degeneration. This limitation is particularly pronounced in proximal and high UN injuries, where delayed reinnervation may preclude meaningful intrinsic muscle recovery.

To address this limitation, the supercharged end-to-side (SETS) anterior interosseous nerve to ulnar motor nerve transfer was introduced.^7^ Initially described in 2009, with early clinical outcomes reported in 2012, subsequent publications have further refined its indications and surgical algorithm.^8^ Given the known axonal regeneration rate of approximately 1–1.5 mm/day, proximal UN injuries require considerable time for regenerating axons to reach the intrinsic hand muscles. During this interval, prolonged denervation predisposes intrinsic muscles to atrophy, fibrosis, and loss of motor endplate viability. The SETS technique aims to “babysit” distal motor endplates by providing an alternative source of innervation, effectively converting a high-level injury into a lower-level one, thereby shortening reinnervation time and improving functional recovery.^9^ Recovery has been evaluated in previous studies using both clinical and electrodiagnostic outcome measures.^10^

Despite the increasing adoption of the SETS AIN-to-ulnar motor nerve transfer for high UN pathology, reported clinical outcomes remain heterogeneous, and the overall effectiveness and safety of the technique have not been clearly established. Accordingly, this systematic review and meta-analysis aimed to systematically evaluate and synthesize the available evidence on outcomes following SETS AIN-to-ulnar motor nerve transfer in patients with UN pathology.

## Materials and Methods

This systematic review and meta-analysis followed a prespecified protocol registered in PROSPERO and was conducted in accordance with Preferred Reporting Items for Systematic reviews and Meta-Analyses guidelines.^11^

### Eligibility criteria

Randomized controlled trials (RCTs) and observational studies reporting postoperative outcomes following SETS AIN-to-ulnar motor nerve transfer for UN pathology were included. SETS was defined as a distal AIN transection with end-to-side coaptation to the ulnar motor fascicles, with the UN left in continuity or concurrently repaired proximally. Studies using alternative terminology (eg, “reverse end-to-side”) were included if the operative construct met this definition. Eligible studies were limited to English language, human investigations. Cadaveric and nonhuman studies, reviews, commentaries, protocols, case reports, and case series with fewer than five patients were excluded, as were studies evaluating alternative nerve transfer techniques.

### Search strategy

A comprehensive literature search was performed using PubMed, MEDLINE, Embase, and the Cochrane Central Register of Controlled Trials (CENTRAL) via the Ovid platform. The final search was conducted on December 10, 2025. The search strategy included the following terms: (“Ulnar nerve” OR “Ulnar neuropathy” OR “Ulnar nerve paralysis” OR “Ulnar nerve injury” OR “Ulnar motor nerve”) AND (“Supercharged” OR “Nerve transfer” OR “End-to-side” OR “End to side” OR “Anterior interosseous nerve” OR “Anterior interosseous transfer” OR “Anterior interosseous” OR “AIN”).

### Study selection and data extraction

Two reviewers independently screened titles, abstracts, and full texts. Data were extracted using a standardized form, with disagreements resolved by consensus or a third reviewer.

Extracted variables included study design, patient demographics, UN pathology, surgical technique, concurrent procedures, postoperative outcomes, follow-up duration, and complications.

### Outcomes

The primary outcome was intrinsic muscle recovery, defined as Medical Research Council (MRC) grade ≥ 3 or ≥ 4 at final follow-up. Secondary outcomes included time to initial intrinsic muscle recovery, functional outcomes (grip strength, key pinch strength, and Disabilities of the Arm, Shoulder and Hand [DASH] scores), residual clinical signs, and postoperative complications.

### Risk of bias and quality assessment

Risk of bias was assessed according to study design using the Newcastle–Ottawa Scale for observational studies, the Joanna Briggs Institute checklist for case series, and the Cochrane Risk of Bias 2 tool for RCTs.^12^, ^13^, ^14^

### Statistical analysis

Random-effects single-arm proportional meta-analyses were performed for binary outcomes. Pooled proportions with 95% CIs were calculated using a random-effects model, and heterogeneity was assessed with the I^2^ statistic. Between-subgroup differences were evaluated using Cochran’s Q test, and subgroup analyses were considered exploratory. Continuous outcomes were summarized descriptively because of heterogeneity in definitions and reporting. Publication bias was not formally assessed because of the limited number of studies per outcome. A two-sided *P* value <.05 was considered statistically significant.

## Results

### Search results and study selection

**Figure 1:**
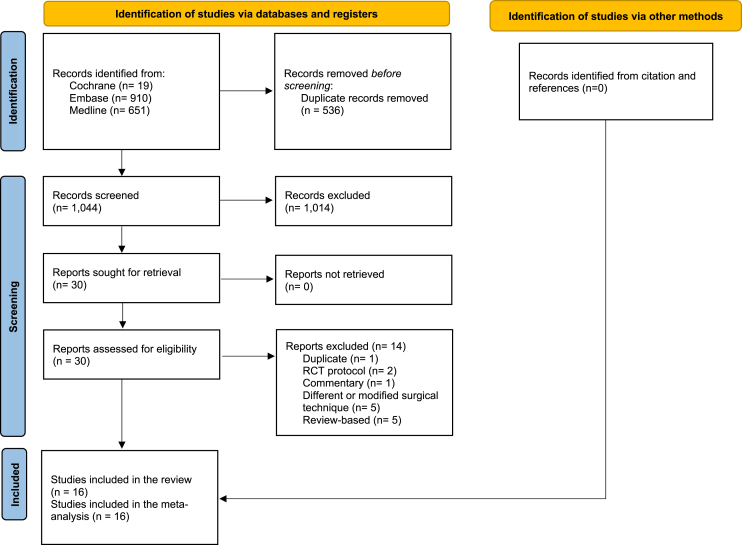
Preferred Reporting Items for Systematic reviews and Meta-Analyses chart of the screening process.

Database searching identified 1,580 records from MEDLINE, Embase, and the Cochrane Central Register of Controlled Trials. After removal of 536 duplicates, 1,044 records were screened, of which 1,014 were excluded. Full texts of 30 studies were assessed, and 14 were excluded for predefined reasons. A total of 16 studies met the inclusion criteria and were included in both the systematic review and meta-analysis (Fig. 1).

### Study characteristics

Sixteen studies published between 2015 and 2025 were included, originating from North America, Asia, the Middle East, and Europe.^2^^,^^10^^,^^15^, ^16^, ^17^, ^18^, ^19^, ^20^, ^21^, ^22^, ^23^, ^24^, ^25^, ^26^, ^27^, ^28^ Most studies were observational, comprising retrospective cohorts and case series, with four prospective cohort studies and one RCT (Table 1).^2^^,^^10^^,^^15^, ^16^, ^17^, ^18^, ^19^, ^20^, ^21^, ^22^, ^23^, ^24^, ^25^, ^26^, ^27^, ^28^

**Table 1:** Summary of Baseline Characteristics of Included Studies

Study Number	Title	First Author, Publication (y)	Country	Study Design	Journal	Level of Evidence
1	Reverse end-to-side anterior interosseous nerve to ulnar motor transfer for severe ulnar neuropathy	Doherty et al^2^ (2020)	Canada	Case series	*Plastic and Reconstructive Surgery*	IV
2	Supercharge end-to-side nerve transfer from anterior interosseous nerve to augment intrinsic recovery in high UN injuries of varying magnitudes	Li et al^15^ (2024)	Taiwan	Prospective cohort	*Asian Journal of Surgery*	III
3	Early and late intrinsic hand muscle reinnervation after end-to-side ain-to-ulnar motor nerve transfer	Mitchell et al^16^ (2025)	Canada	Retrospective cohort	*HAND*	IV
4	Comparison of ulnar intrinsic function following supercharge end-to-side anterior interosseous-to-ulnar motor nerve transfer: a matched cohort study of proximal UN injury patients	Baltzer et al^17^ (2016)	United States and Canada	Retrospective cohort	*Plastic and Reconstructive Surgery*	III
5	Supercharge end-to-side anterior interosseous-to-ulnar motor nerve transfer restores intrinsic function in cubital tunnel syndrome	Dengler et al^10^ (2020)	United States and Canada	Retrospective cohort	*Plastic and Reconstructive Surgery*	IV
6	Morphological characteristics of the cubital tunnel as indication for anterior interosseous nerve supercharge end-to-side transfer in treating advanced cubital tunnel syndrome	Lee et al^18^ (2025)	South Korea	Retrospective cohort	*Orthopaedics & Traumatology: Surgery & Research*	IV
7	UN decompression and transposition with versus without SETS motor nerve transfer for advanced cubital tunnel syndrome: a randomized comparison study	Xie et al^19^ (2022)	China	RCT	*Journal of Neurosurgery*	I
8	SETS anterior interosseous nerve transfer to restore the intrinsic motor function in osteoarthritis-induced cubital tunnel syndrome	Cha et al^20^ (2025)	Korea	Case series	*Journal of Shoulder and Elbow Surgery*	IV
9	End-to-side anterior interosseous nerve transfer: a valuable alternative for traumatic high UN pals	Chen et al^21^ (2021)	Taiwan	Retrospective cohort	*Annals of Plastic Surgery*	IV
10	Comparison between supercharged UN repair by anterior interosseous nerve transfer and isolated UN repair in proximal UN injuries	Koriem et al^22^ (2019)	Egypt	Prospective cohort	*Journal of Hand Surgery*	II
11	Primary repair versus reverse end-to-side coaptation by anterior interosseous nerve transfer in proximal UN injuries	Gontre et al^23^ (2023)	United States	Prospective cohort	*Plastic and Reconstructive Surgery*	III
12	The supercharge end-to-side anterior interosseous-to-ulnar motor nerve transfer for restoring intrinsic function: clinical experience	Davidge et al^24^ (2015)	United States	Retrospective cohort	*Plastic and Reconstructive Surgery*	IV
13	SETS anterior interosseous nerve transfer to restore intrinsic function in high UN injury: a prospective cohort study	Abaskhron et al^25^ (2024)	Egypt	Case series	*BMC Musculoskeletal Disorders*	IV
14	Anterior interosseous nerve transfer combined with cubital and ulnar tunnel release for severe UN compression	Lien et al^26^ (2025)	Taiwan	Prospective cohort	*Journal of Hand Surgery*	IV
15	The hand diagram: A novel outcome measure following SETS anterior interosseous nerve to UN transfer in severe compressive ulnar neuropathy	Knight et al^27^ (2023)	Canada	Retrospective cohort	*Journal of Hand Therapy*	IV
16	Effect of end-to-side anterior interosseous nerve transfer in proximal UN injuries: a comparative study	Yogun et al^28^ (2025)	Turkey	Retrospective cohort	*Journal of Hand and Microsurgery*	III

RCT, randomized controlled trial.

A total of 447 patients were included, with a male predominance (261 men, 111 women) and a mean age of 48.1 years. Compression-related pathology was most common, followed by transection and lesion-in-continuity, with the elbow being the most frequently affected site; reporting of injury subtype and location varied across studies (Table 2).

**Table 2:** Summary of Patient Clinical Characteristics

Study Number	Number of Included Patients/ Operated Nerves	Sex (M: F)	Age (y)	Type of Nerve Pathology, n (%)	Location of Nerve Pathology, n∗ (%)
1	30	21:9	Mean: 53 ± 18.9	Compression: 30	Elbow: 30
2	38	25:13	Mean: 38.8 ± 14.5	Transection: 26 Lesion-in-continuity: 12	Above the elbow: 38
3	17	15:2	Mean: 55± 14	Compression: 14 Transection: 3	Elbow: 14 Upper arm: 3
4	13	NR	Mean: 35 ± 14	Transection: 7 Compression/lesion-in-continuity: 6	Proximal forearm: 5 Elbow: 7 Upper arm: 1
5	42	33:9	Mean: 48	Compression: 42	Elbow: 42
6	43	25:18	Mean: 48.6 ± 10.7	Compression: 43	Elbow: 43
7	45	34:11	Mean: 56	Compression: 45	Elbow: 43 NR: 2
8	27	18:9	Mean: 54 ± 8	Compression: 27	Elbow: 27
9	13	NR	Mean: 38.1	Transection: 13	NR^†^
10	11	NR	NR	Transection: 11	NR
11	32	NR	NR	Transection: 32	Above the elbow: 32
12	55	38:17	Mean: 50.5 ± 15.5	Compression: 23 Lesion-in-continuity: 21 Transection: 7 NR: 4	NR
13	38	25:13	Mean: 32.5 ± 9.1	Compression: 16 Transection: 13 Lesion-in-continuity: 9	Forearm: 5 Elbow: 27 Above the elbow: 6
14	28	20:8	NR	Compression: 28	Elbow: 28
15	9	7:2	Mean: 68 ± 8.6	Compression: 9	Elbow: 9
16	6	NR	NR	Transection: 6	Above the elbow: 6
Total	447	261:111	Mean: 48.1	-	-

F, female; M, male; NR, not reported.

Baseline symptoms were variably reported. Weakness was most common (417 patients, 93.3%), followed by intrinsic muscle atrophy (395 patients, 88.4%). Positive Froment’s sign was observed in 245 patients (54.8%), whereas clawing and numbness were reported in 216 (48.3%) and 185 (41.4%) patients, respectively. Where available, the mean symptom duration was 21.8 months (Table 3).

**Table 3:** Summary of Patient Presenting Symptoms

Study number	Pain, n (%)	Weakness, n (%)	Numbness, n (%)	Intrinsic muscle atrophy, n (%)	Positive Froment’s sign, n (%)	Clawing, n (%)	Wartenberg’s sign, n (%)	Symptom duration (mo)
1	NR	30 (100%)	30 (100%)	30 (100%)	NR	30 (100%)	NR	NR
2	NR	38 (100%)	38 (100%)	38 (100%)	38 (100%)	38 (100%)	38 (100%)	NR
3	NR	17 (100%)	NR	17 (100%)	NR	NR	NR	Mean: 24 ± 28
4	NR	13 (100%)	NR	13 (100%)	NR	NR	NR	NR
5	NR	42 (100%)	NR	42 (100%)	NR	NR	NR	Mean: 31
6	NR	43 (100%)	NR	43 (100%)	NR	NR	NR	Mean: 25 ± 7.8
7	45 (100%)	45 (100%)	45 (100%)	45 (100%)	NR	45 (100%)	NR	Mean: 10.13 ± 3.19
8	NR	27 (100%)	NR	27 (100%)	NR	NR	NR	Mean: 23 ± 5
9	NR	NR	NR	NR	NR	NR	NR	NR
10	11 (100%)	11 (100%)	11 (100%)	11 (100%)	11 (100%)	11 (100%)	NR	Median: 12
11	NR	32 (100%)	NR	32 (100%)	32 (100%)	32 (100%)	NR	Median: 12
12	29 (53%)	55 (100%)	52 (95%)	52 (95%)	45 (82%)	NR	NR	Mean: 33.6 ± 57.0
13	NR	38 (100%)	NR	36 (94.7%)	38 (100%)	33 (86.8%)	33 (86.8%)	Mean: 6.16 ± 3.14
14	NR	28 (100%)	NR	NR	28 (100%)	27 (96.4%)	27 (96.4%)	Median: 12 (IQR: 3–36)
15	NR	9 (100%)	9 (100%)	9 (100%)	8 (89%)	NR	NR	NR
16	NR	NR	NR	NR	NR	NR	NR	NR
Total	85 (19%)	417 (93.3%)	185 (41.4%)	395 (88.4%)	245 (54.8%)	216 (48.3%)	98 (21.9%)	Mean: 21.8

IQR, interquartile range; NR, not reported.

Reporting of time from symptom onset to surgery was heterogeneous, with a mean of 22.7 months among studies providing data. The mean follow-up duration was 19.3 months, with variability in follow-up length and completeness (Table 4).

**Table 4:** Summary of Surgical Details and Follow-up Duration

Study Number	Time From Symptoms-Onset to Surgery (mo)	Concurrent Procedures, n (%)	Follow-up Duration (mo)
1	NR	Subcutaneous UN transposition: 30 (100%)	Mean: 18.6 ± 7.7
2	Mean: 8.5 ± 2.3	Ulnar and cubital tunnel release: 38 (100%) Subcutaneous anterior transposition: 17 (44.7%)	Mean: 23.5 ± 8.2 (only 30 patients were followed up)
3	Mean: 24 ± 28	Guyon canal release: 17 (100%) Anterior UN transposition: 14 (82.4%)	Mean: 17.3 ± 3.6
4	Mean: 4.4 ± 3.5	Guyon canal decompression: 6 (46.2%) Neurolysis: 6 (46.2%) Ulnar nerve repair: 7 (53.8%) Cubital tunnel decompression: 6 (46.2%)	Mean: 13.5 ± 13
5	Mean: 31	Ulnar nerve transposition: 34 (81.0%) Guyon canal release: 40 (95.2%) Carpal tunnel release: 35 (83.3%) Sensory cross-palm grafts: 30 (71.4%) Profundus tenodesis: 31 (73.8%)	Mean: 11.2 ± 7.6
6	Mean: 25 ± 7.8	Cubital tunnel release: 43 (100%)	Range: 6–12
7	NR	Cubital tunnel decompression with anterior subfascial transposition: 45 (100%)	Mean: 27.70 ± 2.18
8	NR	Cubital tunnel decompression: 27 (100%) Guyon tunnel decompression: 27 (100%) Ulnar nerve transposition: 15 (55.6%)	Mean: 24
9	Mean: 71.5	NR	NR
10	NR	NR	Mean: 18
11	NR	Primary ulnar nerve epineural repair: 32 (100%) Guyon canal decompression: 32 (100%)	Mean: 12
12	Mean: 33.6 ± 57.0	Guyon canal decompression: 55 (100%) Profundus tenodesis: 37 (67.3%) Primary ulnar nerve repair: 26 (47.3%) Carpal tunnel release: 28 (50.9%) Tendon transfers, 8 (14.5%) Pronator release: 4 (7.3%)	Mean: 8.0 ± 5.7 (only 39 patients were followed up)
13	Mean: 6.16 ± 3.14	Anterior ulnar nerve transposition: 38 (100%) Direct ulnar nerve repair, 14 (36.8%) Ulnar nerve grafting: 3 (7.9%)	Mean: 18
14	Median: 12 (IQR: 3–36)	NR	Mean: 24
15	NR	NR	Mean: 22.8 ± 9.3
16	Mean: 0.02 ± 0.01	NR	Mean: 31.17 ± 10.69
Total	Mean: 22.7	-	Mean: 19.3

IQR, interquartile range; NR, not reported; n (%), number (percentage).

### Risk of bias assessment

Overall, the included studies demonstrated predominantly low to moderate risk of bias. Among the nine observational comparative and cohort studies, eight were classified as low risk of bias, whereas one study, Chen et al^21^, was judged to have moderate risk because of its retrospective design and limited reporting on cohort selection and follow-up.

Of the eight case series, two studies, Li et al^15^ and Lien et al^26^, demonstrated low risk of bias, whereas six were judged to have moderate risk primarily because of retrospective design, incomplete case inclusion, and insufficient reporting of follow-up.

The single RCT by Xie et al^19^ was judged to have some concerns, reflecting inherent challenges in surgical trials.

### Intrinsic muscle recovery

**Figure 2:**
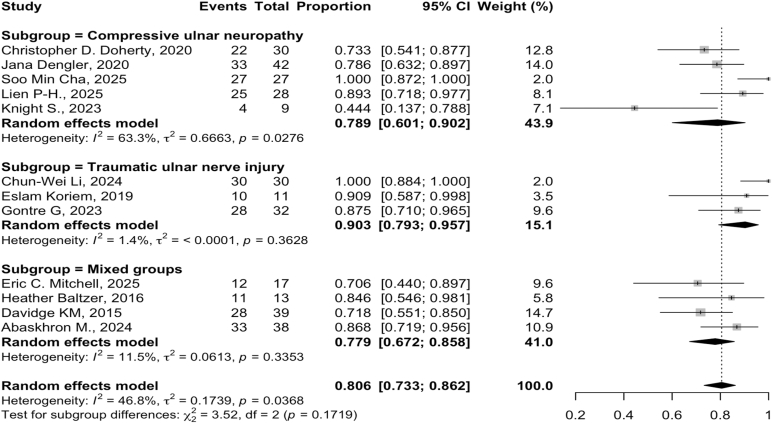
Meta-analysis of clinical evidence for intrinsic muscle recovery or reinnervation (MRC grade ≥ 3) at final follow-up, with subgroup analysis based on the pathology type (compressive ulnar neuropathy, traumatic UN injury, or mixed groups).

Clinical evidence of intrinsic muscle recovery was reported in most studies and was most commonly assessed using the first dorsal interosseous muscle, either alone or in combination with other ulnar-innervated intrinsic muscles, such as the abductor digiti minimi (Table 5). The pooled proportion of patients achieving intrinsic muscle recovery, defined as MRC grade ≥ 3, at final follow-up was 80.6% (95% CI 73.3–86.2), with moderate heterogeneity (I^2^ = 46.8%) (Fig. 2).

**Table 5:** Summary of Assessment Methods for Postoperative Outcomes

Study Number	Muscles Assessed for Intrinsic Muscle Recovery or Reinnervation Grading Using the MRC^¶^ Scale	Time to First Intrinsic Muscle Recovery/Reinnervation (mo)	Definition of First Intrinsic Muscle Recovery or Reinnervation
1	FDI and ADM	Mean: 8.5 ± 4.9	Clinical and/or electrophysiologic
2	FDI	Mean: 8.8 ± 5.9	Clinical
3	FDI and ADM	Mean: 10.7 ± 4	Clinical and/or electrophysiologic
4	FDI	Mean: 2.9 ± 1.4	Clinical
5	FDI	<1 mo (5.1%), 1–3 mo (48.7%), 3–15 mo (46.2%)	Clinical
6	FDI and ADM	NR	Electrophysiologic
7	FDI and ADM	NR	Clinical
8	FDI	NR	Clinical
9	NR	NR	NR
10	Ulnar intrinsic muscle	Range: 6–12	Clinical and/or electrophysiologic
11	FDI and ADM	Mean: 6	Clinical and/or electrophysiologic
12	FDI	NR	Clinical
13	FDI and FDP	Mean: 6.85 ± 1.3	Clinical
14	FDI	Median: 9 (IQR: 6–12)	Clinical
15	FDI and ADM	Mean: 8.5	Clinical
16	FDI, ADM, FCU, and FDP	Mean: 31	Clinical and/or electrophysiologic
Total	-	Mean: 10.4	-

ADM, abductor digiti minimi; FCU, flexor carpi ulnaris, FDP, flexor digitorum profundus; FDS, first dorsal interosseous; IQR, interquartile range; NR, not reported.

**Figure 3:**
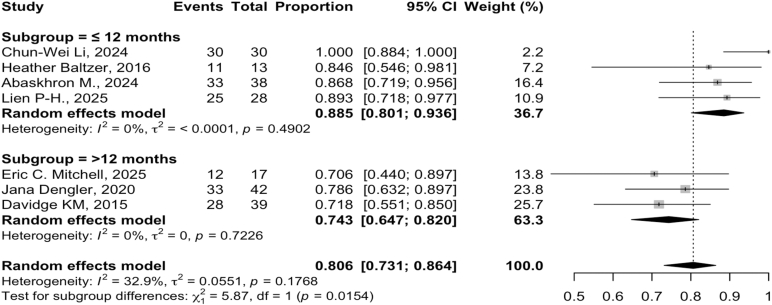
Meta-analysis of clinical evidence for intrinsic muscle recovery or reinnervation (MRC grade ≥ 3) at final follow-up, with subgroup analysis based on the timing of intervention (≤12 months versus >12 months).

Pathology-based subgroup analysis for MRC grade ≥ 3 recovery demonstrated pooled recovery proportions of 78.9% in compressive ulnar neuropathy, 90.3% in traumatic UN injuries, and 77.9% in mixed cohorts, with no statistically significant difference noted between groups (*P* = .1719; Fig. 2). When stratified by timing of intervention, surgery performed within 12 months of symptom onset was associated with a higher pooled proportion of intrinsic recovery compared with surgery performed more than 12 months after symptom onset (*P* = .0154) (Fig. 3).

**Figure 4:**
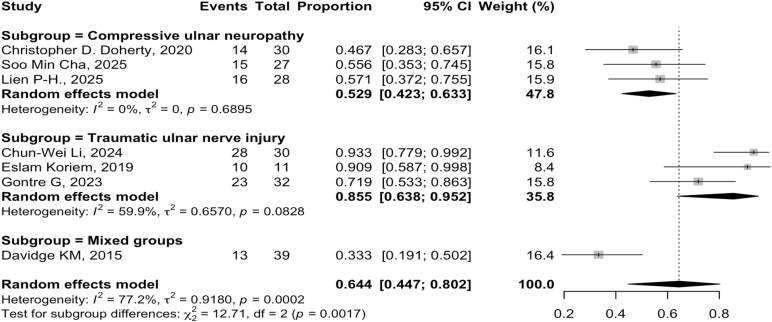
Meta-analysis of clinical evidence for intrinsic muscle recovery or reinnervation (MRC grade ≥ 4) at final follow-up, with subgroup analysis based on the pathology type (compressive ulnar neuropathy, traumatic ulnar nerve injury, or mixed groups)

Advanced intrinsic muscle recovery, defined as near-normal strength (MRC grade ≥ 4), was reported less frequently across studies. Recovery was primarily assessed in the first dorsal interosseous muscle, with some studies additionally evaluating the abductor digiti minimi and other ulnar-innervated intrinsic muscles (Table 5). The pooled proportion of patients achieving MRC grade ≥ 4 recovery at final follow-up was 64.4% (95% CI 44.7–80.2), with substantial heterogeneity (I^2^ = 77.2%; Fig. 4).

**Figure 5:**
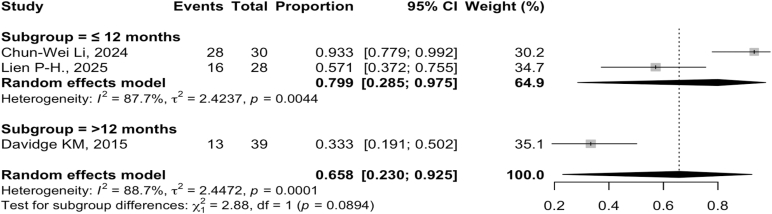
Meta-analysis of clinical evidence for intrinsic muscle recovery or reinnervation (MRC grade ≥ 4) at final follow-up, with subgroup analysis based on the timing of intervention (≤12 months versus >12 months).

Subgroup analysis for MRC grade ≥ 4 recovery demonstrated pooled recovery proportions of 52.9% in compressive ulnar neuropathy, 85.5% in traumatic UN injuries, and 33.3% in mixed cohorts, with a statistically significant difference favoring traumatic injuries (*P* = .0017; Fig. 4). When stratified by surgical timing, intervention within 12 months demonstrated a higher pooled proportion of advanced recovery compared with intervention beyond 12 months, although this difference did not reach statistical significance (*P* = .0894; Fig. 5).

### Time to intrinsic muscle reinnervation

Time to intrinsic muscle recovery was reported heterogeneously across studies and was not pooled quantitatively. Definitions varied and included clinical detection of muscle contraction, electrophysiologic evidence of reinnervation, or both. Reported mean recovery times ranged widely, from as early as 2.9 months to as late as 31 months, likely reflecting differences in outcome definitions, patient characteristics, chronicity of denervation, and study design. Electrophysiologic assessments generally have identified reinnervation earlier than clinical examination, whereas delayed recovery times may have reflected late clinical detection or prolonged denervation prior to surgery. Accordingly, the unweighted mean time of approximately 10.4 months should be interpreted with caution and considered a descriptive estimate rather than a precise clinical benchmark. In addition, the first dorsal interosseous and abductor digiti minimi muscles were most commonly used to define initial recovery (Table 5).

### Patient-reported and objective functional outcomes

Functional outcomes were summarized descriptively because of heterogeneity in reporting (Table 6). Among studies reporting mean values, the unweighted mean DASH score at final follow-up was 25.3, indicating mild to moderate residual upper limb disability. Mean grip strength averaged 75.7% of the contralateral side, with a mean relative improvement of 70.2% from baseline. Key pinch strength averaged 60.1% of the contralateral side, corresponding to a mean relative improvement of 54.6%.

**Table 6:** Summary of Postoperative Functional and Quantitative Outcomes

Study Number	DASH Score at the Last Follow-up	Grip Strength Compared to the Unaffected/Contralateral Side at the Last Follow-up (% of contralateral)	Grip Strength Improvement Relative to the Preoperative Status at the Last Follow-up (% change from baseline)	Key Pinch Strength Compared to the Unaffected/Contralateral Side at the Last Follow-up (% of contralateral)	Key Pinch Strength Improvement Relative to the Preoperative Status at the Last Follow-up (% change from baseline)
1	NR^†^	NR	NR	NR	NR
2	Mean: 32 ± 7.5	Mean: 82% ± 17.5%	Mean: 126%	NR	Mean: 125.6%
3	NR	NR	NR	NR	NR
4	NR	Mean: 62% ± 30%	NR	NR	NR
5	Mean: 28 (IQR: 12–36)	NR	Mean: 13.8%	NR	Mean: 28.6%
6	Mean: 15.6 ± 5.8	Mean: 59.7% ± 4.7%	Mean: 69.1%	NR	NR
7	Mean: 12.9 ± 4.4	Mean: 81.3% ± 9.7%	Mean: 127.4%	Mean: 75.1% ± 7.7%	Mean: 114.9%
8	Mean: 20 ± 3	Mean: 83% ± 7%	Mean: 22.1%	Mean: 84% ± 7%	Mean: 25.4%
9	NR	NR	NR	NR	NR
10	NR	NR	NR	NR	NR
11	Mean: 24.0 ± 3.1	Mean: 93.9% ± 25.9%	Mean: 53.6%	Mean: 14.4% ± 3.8%	Mean: 20%
12	Mean: 38.3 ± 19.1	NR	Mean: 29.6%	NR	Mean: 29%
13	Mean: 9.7 ± 10.8	NR	Mean: 120.0%	NR	NR
14	Median: 5 (IQR: 3–13)	Median: 94% (IQR: 76%–102%)	NR	Median: 65% (IQR: 52%–87%)	NR
15	Mean: 33 ± 28.7	Mean: 81% ± 10.5%	NR	Mean: 72% ± 19.3%	Mean: 38.5%
16	Mean: 39.6 ± 9.3	Mean: 63% ± 11%	NR	Mean: 55% ± 17%	NR
Total	Mean: 25.3	Mean: 75.7%	Mean: 70.2%	Mean: 60.1%	Mean: 54.6%

IQR, interquartile range; NR, not reported.

### Residual Ulnar Nerve-Related Clinical Signs

**Figure 6:**
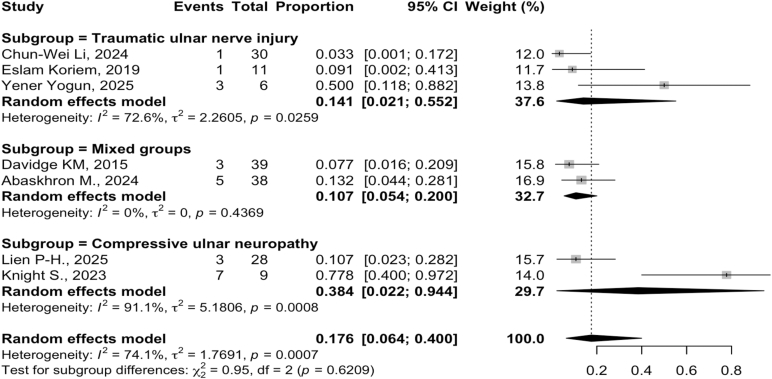
Meta-analysis of the presence of Froment’s sign at final follow-up, with subgroup analysis based on the pathology type.

Persistent Froment’s sign at final follow-up was reported across all pathology subgroups. The pooled prevalence was 17.6%, with substantial heterogeneity (I^2^ = 74.1%) (Fig. 6). Subgroup prevalence values were 38.4% for compressive ulnar neuropathy, 14.1% for traumatic UN injury, and 10.7% for mixed cohorts, with no statistically significant difference between groups (*P* = .6209).

**Figure 7:**
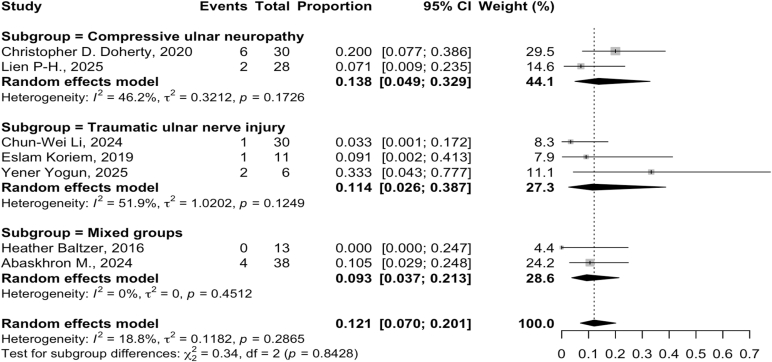
Meta-analysis of ulnar clawing prevalence at final follow-up, with subgroup analysis based on the pathology type.

Residual ulnar clawing was reported in a subset of studies, with a pooled prevalence of 12.1% at final follow-up and low to moderate heterogeneity (I^2^ = 18.8%) (Fig. 7). Subgroup prevalences were 13.8% in compressive ulnar neuropathy, 11.4% in traumatic UN injury, and 9.3% in mixed cohorts, with no statistically significant difference between groups (*P* = .8428).

**Figure 8:**
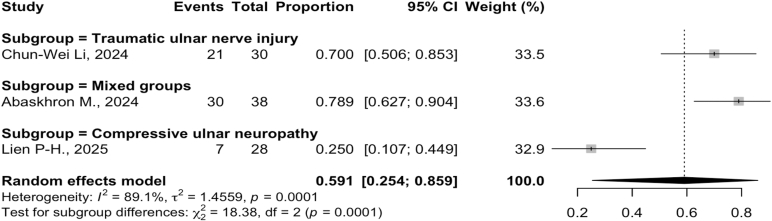
Meta-analysis of Wartenberg’s sign prevalence at final follow-up, with subgroup analysis based on the pathology type.

Wartenberg’s sign demonstrated a pooled prevalence of 59.1%, with considerable heterogeneity (I^2^ = 89.1%) (Fig. 8). Subgroup analysis showed prevalences of 25.0% in compressive ulnar neuropathy, 70.0% in traumatic UN injury, and 78.9% in mixed cohorts, with a statistically significant difference between groups (*P* = .0001).

**Figure 9:**
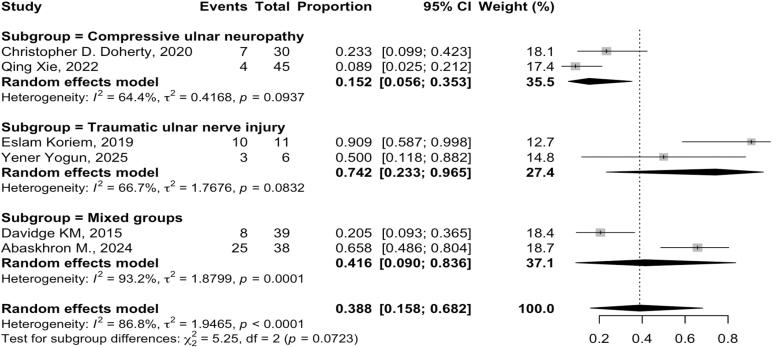
Meta-analysis of intrinsic muscle wasting/atrophy prevalence at final follow-up, with subgroup analysis based on the pathology type.

Persistent intrinsic muscle wasting or atrophy at final follow-up had a pooled prevalence of 38.8%, with high heterogeneity (I^2^ = 86.8%) (Fig. 9). Subgroup prevalences were 15.2% in compressive ulnar neuropathy, 74.2% in traumatic UN injury, and 41.6% in mixed cohorts. The difference between groups did not reach statistical significance (*P* = .0723); however, a trend toward higher prevalence in traumatic injuries was observed.

### Overall complications

**Figure 10:**
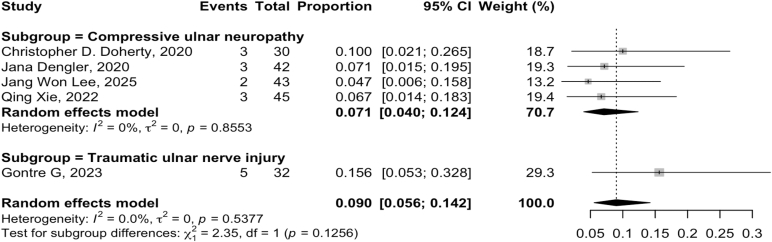
Meta-analysis of overall complications during the follow-up period, with subgroup analysis based on the pathology type.

Postoperative complications were reported in a subset of studies and included wound-related issues, transient neuropathic symptoms, and procedure-related adverse events, with no consistent reports of catastrophic complications. The pooled prevalence of complications was 7.9%, with no observed heterogeneity (I^2^ = 0%) (Fig. 10). Subgroup analysis demonstrated pooled complication rates of 7.1% for compressive ulnar neuropathy and 9.0% for traumatic UN injuries, with no statistically significant difference between groups (*P* = .1256).

## Discussion

To the best of our knowledge, the present systematic review and meta-analysis represent the most comprehensive quantitative synthesis to date of clinical outcomes following SETS AIN-to-ulnar motor nerve transfer for UN pathology. The results suggest that SETS is an effective technique, achieving a pooled functional recovery rate of 80.6% (MRC grade ≥ 3) with a low complication rate of 7.9%, supporting consideration of its incorporation into modern treatment algorithms for high-level UN injuries and severe compressive neuropathy. Compared with outcomes reported for high UN lesions treated with conventional repair or grafting alone, this recovery rate represents a meaningful improvement. Higher-level UN injuries, particularly those proximal to the flexor digitorum profundus, are associated with poor functional prognosis.^5^ The SETS technique mitigates this limitation by providing a distal source of innervation, thereby shortening reinnervation time and preserving motor endplates.^7^^,^^9^ The moderate heterogeneity observed (I^2^ = 46.8%) likely reflects variability in surgical technique, surgeon experience, follow-up duration, and outcome assessment. Notably, recovery rates were consistent across pathology subgroups (compressive 78.9%, traumatic 90.3%, and mixed 77.9%), suggesting that endplate preservation may operate independently of injury etiology.

Subgroup analysis demonstrated that intervention within 12 months of symptom onset was associated with superior functional recovery (MRC ≥ 3) compared with delayed intervention (*P* = .0154). This finding aligns with established timelines of motor endplate degeneration, in which prolonged denervation beyond 12–18 months results in progressive Schwann cell atrophy, muscle fiber loss, and endplate receptor downregulation.^4^^,^^29^ The clinically relevant 12-month threshold challenges prolonged conservative management strategies for compressive neuropathies. The findings support timely surgical intervention when clinical or electrodiagnostic evidence indicates notable nerve damage or inadequate spontaneous recovery. Nevertheless, the mean symptom duration across included studies was 21.8 months, highlighting substantial delays in diagnosis and referral and underscoring the need for improved awareness and clearer surgical indications.

Although functional recovery (MRC ≥ 3) was comparable across pathology subgroups, advanced recovery (MRC ≥ 4) differed considerably, favoring traumatic injuries (85.5%) over compressive neuropathy (52.9%) and mixed pathology (33.3%) (*P* = .0017). This disparity may reflect younger patient age, shorter denervation periods, and less chronic scarring in traumatic injuries compared with compression neuropathy, which typically evolves over time.^30^^,^^31^ Additionally, traumatic injuries often prompt earlier surgical intervention, whereas compressive neuropathies frequently undergo prolonged conservative treatment. Lower recovery rates in mixed cohorts likely represent salvage presentations with more advanced pathology or longer symptom duration. These findings have important implications for patient counseling and refinement of indication criteria.

The unweighted mean time to first intrinsic muscle recovery was 10.4 months, representing a substantial improvement relative to conventional proximal repairs. Given the axonal regeneration rate of approximately 1–1.5 mm per day, proximal UN injuries require prolonged periods for regenerating axons to reach intrinsic hand muscles.^8^ By effectively converting a high-level injury into a lower-level injury, SETS shortens reinnervation time and improves functional recovery.^8^^,^^9^ Variability in reported recovery times reflects heterogeneity in outcome definitions because electrophysiologic assessments typically detect reinnervation earlier than clinical examination.^32^

Functional outcomes provide important context for interpreting motor recovery. The mean DASH score of 25.3 indicates mild to moderate residual upper limb disability, reflecting incomplete restoration of preinjury function. Grip strength recovery (75.7% of the contralateral side) exceeded key pinch recovery (60.1%), highlighting the critical contribution of intrinsic muscles to fine hand function. However, restoration of intrinsic strength remains challenging with any reconstructive strategy.^6^ The observed improvements from baseline (grip 70.2%, pinch 54.6%) represent clinically meaningful gains that facilitate return to daily and occupational activities.

Evaluation of residual clinical signs further illustrates the limitations of recovery. The low prevalence of persistent Froment’s sign (17.6%) and ulnar clawing (12.1%) suggests effective restoration of thumb adduction and metacarpophalangeal extension. In contrast, Wartenberg’s sign persisted in 59.1% of patients, with a notably higher prevalence in traumatic and mixed pathology groups compared with compressive neuropathy (*P* = .0001). The high prevalence of persistent Wartenberg’s sign despite recovery of intrinsic strength may reflect differential reinnervation patterns among ulnar-innervated muscles, with incomplete restoration of small finger abduction control. Additionally, chronic denervation changes, intrinsic muscle imbalance, and altered tendon mechanics may contribute to persistent clinical signs despite measurable improvements in motor strength. Similar differential recovery patterns have been reported previously.^16^ Persistent intrinsic muscle atrophy was observed in 38.8% of patients despite functional strength recovery in most cases, highlighting that restoration of strength and muscle bulk follow distinct biological trajectories.^4^

The pooled complication rate of 7.9%, with no observed heterogeneity (I^2^ = 0%), supports the safety of SETS across institutions and pathology subgroups.^10^^,^^24^ Donor-site morbidity from AIN harvest appears to be minimal, consistent with prior experimental and clinical studies of reverse end-to-side nerve transfer techniques.^7^ Taken together, the favorable risk–benefit profile supports SETS as a valuable addition to the reconstructive armamentarium for UN pathology.

Several limitations warrant consideration. Most included studies were retrospective observational designs, with only one RCT identified.^19^ Heterogeneity in outcome definitions, assessment methods, and follow-up duration precluded quantitative pooling of continuous outcomes, and the absence of comparator groups limits conclusions regarding superiority over conventional repair or grafting. All subgroup analyses were exploratory and based on nonrandomized data. The mean follow-up duration of 19.3 months may also be insufficient to assess long-term durability. Future studies should prioritize prospective, multicenter designs with standardized outcome measures and follow-up intervals. Development of core outcome sets and registry-based comparative studies may help address remaining questions regarding optimal technique, interaction with concurrent procedures, cost-effectiveness, and patient-reported outcomes beyond DASH.

An important limitation is the inclusion of heterogeneous UN pathologies, including both compressive neuropathy and traumatic injuries, which differ in mechanism, chronicity of denervation, and expected recovery potential. Although subgroup analyses were performed based on pathology type (compressive, traumatic, and mixed cohorts), pooled estimates should be interpreted as reflecting outcomes across mixed etiologies rather than a single uniform clinical population.

Although subgroup analysis suggested improved recovery with intervention within 12 months, this finding should be interpreted cautiously. Timing of surgery is likely influenced by multiple factors, including injury severity, duration of denervation, surgeon preference, and the use of concomitant procedures such as decompression or transposition. As such, the observed association may not reflect a purely causal relationship.

Additionally, outcome assessment was not standardized across studies. Although the intrinsic muscles evaluated are detailed in Table 5, reporting of the assessor (treating surgeon versus independent examiner) and follow-up time points was inconsistent or absent in several studies, limiting comparability of reported recovery outcomes.

The wide variability in reported recovery times further limits the interpretability of this outcome. Extreme values may reflect differences in detection methods, with electrophysiological studies identifying early reinnervation prior to clinically observable contraction, whereas delayed recovery times may be influenced by prolonged denervation, late presentation, or variability in follow-up intervals. As such, recovery timelines should be interpreted as approximate and context-dependent rather than fixed expectations in clinical practice.

This meta-analysis demonstrates that SETS AIN-to-ulnar motor nerve transfer is an effective and safe technique for restoring intrinsic hand function in patients with UN pathology, achieving functional recovery in 80.6% of patients with a low complication rate of 7.9%. Intervention within 12 months of symptom onset is associated with improved outcomes, emphasizing the importance of timely surgical decision making in appropriately selected patients. Although traumatic injuries demonstrate higher rates of near-normal recovery than compressive neuropathies, clinically meaningful functional gains are observed across all pathology subgroups. The current evidence supports consideration of SETS within treatment algorithms for high UN lesions and severe compressive neuropathy, while highlighting the need for prospective studies with standardized outcome measures to refine patient selection, optimize technique, and establish long-term durability.

## Conflicts of Interest

No benefits in any form have been received or will be received related directly to this article.
